# When to look at maps in navigation: metacognitive control in environment learning

**DOI:** 10.1186/s41235-018-0130-7

**Published:** 2018-09-26

**Authors:** Ruizhi Dai, Ayanna K. Thomas, Holly A. Taylor

**Affiliations:** Department of Psychology, Tufts University, 490 Boston Avenue, Medford, MA 02155 USA

**Keywords:** Environment learning, Perspective switching, Metacognitive control

## Abstract

People learn environments through direct experience with the environment and/or through map study. Further, the different perspectives taken while learning an environment influence the knowledge acquired. After all, different information about an environment is readily available through route (e.g. by navigation) and survey (e.g. with maps) perspectives. Having a choice between direct experience and map use, or between different perspectives, suggests a role of metacognitive control in environment learning. That is, when in a new environment, learners may exercise metacognitive control by selectively choosing and implementing specific learning strategies, such as switching between perspectives. Strategy choice may depend on specific constraints, such as perspective, range of view, or amount of time to learn (to name a few). For example, people may check a map (e.g. on smartphones or GPS devices) to complement developing route knowledge. The present review discusses the role of metacognition in environment learning and outlines new directions for research to bridge these fields by examining how strategic metacognitive control over perspective switching affects environment learning. Such explorations can inform real-world environment learning and navigational aids design.

## Significance

When people take either a survey (e.g. study a map) or a route perspective (e.g. navigate) while learning an unfamiliar environment, their spatial knowledge differs. However, in the real world, people are not generally restricted to one perspective or the other when learning new environments. With navigational aids, such as smartphones and devices using Global Positioning System (GPS), we can flexibly choose and switch between route and survey perspectives, rather than taking only one perspective. Although studies have explored how switching perspectives may affect environment learning (EL), the metacognitive aspect of perspective switching is presently poorly understood. Exploration of metacognitive processes in EL would allow us to better understand spatial knowledge development, to potentially target and facilitate real-world learning of unfamiliar environments. Further, it could provide insights into navigational aid design. Considering findings from EL and metacognitive control literature together, the present paper outlines new directions to bridge these fields and calls for studies to explore the role metacognition plays in EL.

## Introduction

Imagine that you have just moved to a new neighborhood and are trying to learn the environment. What will you do? Would you try to navigate within the neighborhood to learn routes and landmarks, study the information from a map, or take a walk with the help of your smartphone?

**Fig. 1:**
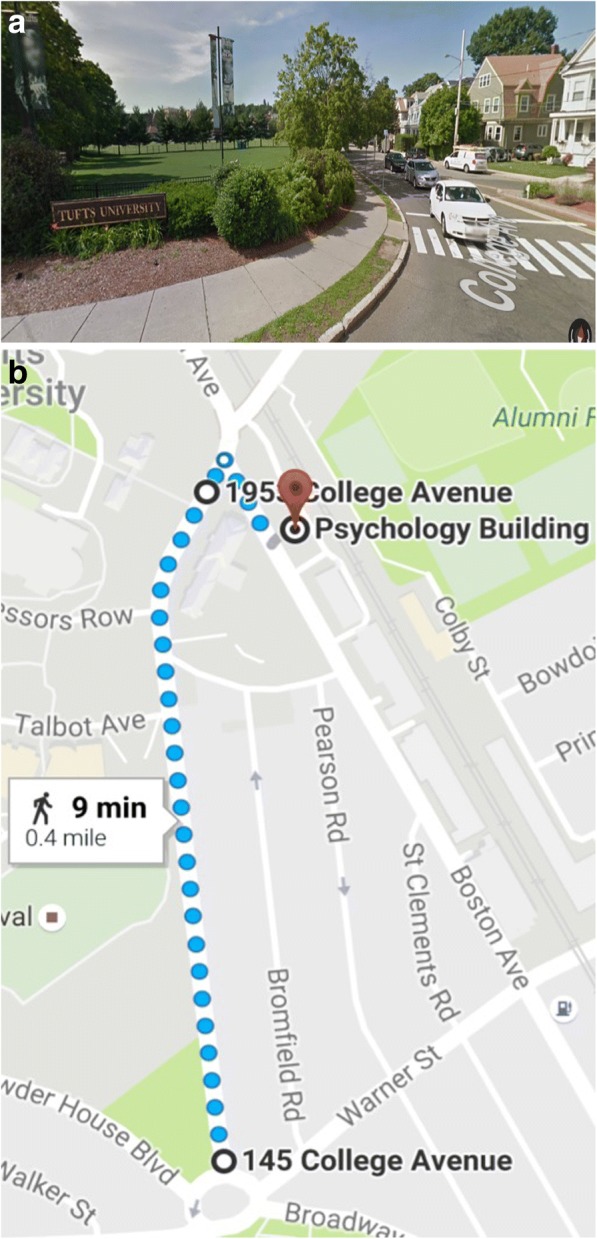
**a** An example of route perspective display (Google Maps, 2018a). **b** An example of survey perspective display (Google Maps, 2018b)

Environment learning (EL) is a process in which individuals encode spatial information (such as the spatial relations between locations) within an environment. When learning a new environment, people typically take either a route perspective, which is a person-centered view from inside the environment, or a survey perspective, which is a map-like, top-down view (Linde & Labov, 1975; Thorndyke & Hayes-Roth, 1982). Previous research investigating EL suggests that the knowledge or representation developed may differ based on the perspective taken during learning (Schneider & Taylor, 1999; Taylor & Tversky, 1992; Thorndyke & Hayes-Roth, 1982). However, active learners often alternate among different spatial information sources, including ground-level, first-person navigation within the environment (route perspective, see Fig. 1a), and navigational aids, like maps on mobile devices (survey perspective, see Fig. 1b). Modern technology, such as GPS and smartphones, has made it easier to switch between route and survey perspectives. For example, you might check your location on a smartphone when feeling lost in your new neighborhood. Although understudied in the environmental learning domain, these anecdotal observations suggest a role of metacognition in navigation.

Metacognition, and specifically metacognitive control, involves the regulation or control of the learning process (Nelson & Narens, 1990, 1994). Predominantly within the verbal learning literature, research has demonstrated that learners tend to control what, when, and how to study; they also choose strategies to presumably enhance learning (Kornell & Finn, 2016; Kornell & Metcalfe, 2006; Tullis & Benjamin, 2012). In this review, we discuss the role of metacognitive control in EL. Towards this end, we first introduce theoretical models of metacognition, discuss previous literature on perspective switching in EL, and review factors that influence metacognitive control and EL. We then focus on research questions to explore the possible relationship between metacognitive control and EL. We hypothesize that strategic control over perspective switching could affect spatial knowledge development. Future directions and possible applications are discussed.

## Review

### Metacognition: monitoring and control

Metacognition, usually described as “knowing about knowing” (Metcalfe & Shimamura, 1994) and “cognition about cognition” (Flavell & Ross, 1981), is commonly defined as the beliefs or knowledge about our cognition, and the regulation or control of cognition and learning (Allen & Armour-Thomas, 1991; Cross & Paris, 1988; Flavell & Ross, 1981; Schraw, 1998).

Nelson and Narens (1990, 1994) proposed that metacognition can be considered as two interacting components: (1) metacognitive monitoring, or consciously assessing learning and/or later retrievability; and (2) metacognitive control, or strategically regulating learning by implementing specific strategies. One dominant model of metacognition suggests that monitoring and control enable dynamic interactions between metacognitive and cognitive functions, in that metacognitive processes are informed by, and also modify cognitive processes (Nelson, 1996; Nelson & Narens, 1990, 1994; see also Winne, 2001). Further, research consistently demonstrates that monitoring affects control by, for example, influencing how participants allocate study time to reduce the discrepancy between a desired learning goal and the present state of learning (Dunlosky & Hertzog, 1998; Metcalfe & Kornell, 2005). For instance, learners allocate more time to items perceived as difficult, as these likely show the largest discrepancies between the present and desired state (Koriat, Ma’ayan, & Nussinson, 2006; Metcalfe & Kornell, 2005). However, when study time is limited, learners devote more time to easier as opposed to difficult items, and choose easier items for restudy (Dunlosky & Hertzog, 1998; Kornell & Finn, 2016; Son & Metcalfe, 2000; Thiede & Dunlosky, 1999). Regardless of how regulation or control is implemented, this body of research demonstrates how metacognitive monitoring and control interact to strategically regulate learning.

Another model addressing the relationship between metacognitive monitoring and control comes from self-regulated learning research. According to Pintrich (2000), self-regulated learning is a process in which learners set learning goals, monitor cognition, and control learning behaviors based on established goals and environmental features (Pintrich, 2000, p. 453). Pintrich described self-regulated learning as consisting of four primary elements: (1) *planning*, which includes target goal setting and activation of perceptions and prior knowledge; (2) *monitoring*, including metacognitive awareness and monitoring of current cognition; (3) *controlling and regulating* study by selecting cognitive strategies for learning and thinking; and (4) *reacting and reflecting* on the task (Pintrich, 2004; Schraw, Crippen, & Hartley, 2006). Within this framework, self-regulated learners actively use a variety of strategies such as planning, organizing, self-monitoring, and self-evaluating to facilitate learning (Zimmerman, 1986). While these phases overlap with some of the processes proposed in Nelson and Naren’s framework, Pintrich’s model emphasizes learners’ autonomy in strategically engaging control of learning, as it highlights individuals’ active role in monitoring and control (Pintrich, 2000).

Whereas metacognition has been carefully investigated within the context of verbal learning, research extending this examination to non-verbal domains is sparse at best. It is reasonable to assume that metacognitive processes predicted to dynamically interact with cognition, should extend to cognitive processes that underlie EL. Returning to our earlier example, when learning your new neighborhood, choices you make likely impact navigation and route learning. Based on Nelson and Narens (1994), being able to both accurately monitor how well you have learned the new neighborhood and select strategies to facilitate learning, should affect how you study the new neighborhood. Specifically, when feeling lost in the new neighborhood (monitoring), you may refer to a digital map on your mobile device to figure out where to go next (control). Your learning outcome is determined by the accuracy of monitoring and the effectiveness of control. Pintrich’s model (2000) would predict that in self-regulated EL, you may actively plan ahead of time by setting up a study goal, e.g. study a map to later freely navigate in the environment. You may then closely monitor your learning, for example by constantly asking yourself about landmark locations. The outcome of this monitoring may then lead you to select strategies for better learning. After then navigating through the environment, you may reflect on your performance, thinking of ways to further improve your memory for the neighborhood.

Consideration of different metacognition frameworks allows researchers to make different predictions regarding metacognition in navigational control, as well as the relationship between monitoring and control in navigation. We discuss how future research may capitalize on these considerations at the end of this review.

### Environment learning: different spatial perspectives

Environment learning can be achieved through map study or through direct experience with the environment, such as navigating between locations. People usually engage in EL in a goal-directed manner. For instance, when learning maps or navigating, individuals may have two main goals: to facilitate navigation between locations; or to remember the layout and the routes between landmarks. These different goals may affect the type of strategies adopted, further influencing what and how information is learned (Taylor, Naylor, & Chechile, 1999).

Research has suggested that learning from maps or from navigation differs in many aspects. Maps usually show the environment’s overall layout, from a survey perspective or bird’s eye viewpoint. With maps, the reference is object-centered; while maps can be rotated, the orientation is usually stable (e.g. north-up). Through navigation, however, people experience the environment from their own first-person perspective. The reference system is viewer-centered and the orientation changes as the navigator turns. Additionally, maps and navigation emphasize different types of spatial relations. With maps, because they present an overview of the environment, all landmark-to-landmark relations can be viewed directly. In contrast, when navigating usually only part of the environment can be seen directly. Thus, while some landmark-to-landmark relations can be learned directly while navigating, it is limited to those in the current visual field. Instead, self-to-landmark relations are readily apparent. In general, these differences demonstrate the distinction between survey (e.g. map learning) and route (e.g. navigation) perspectives (Thorndyke & Hayes-Roth, 1982).

Learning perspective impacts the spatial knowledge acquired (Evans & Pezdek, 1980; Sholl, 1987; Taylor et al., 1999; Thorndyke & Hayes-Roth, 1982). For instance, Thorndyke and Hayes-Roth (1982) found that map learners more accurately estimated global spatial relations and Euclidian (straight-line) distance between locations; however, navigators more accurately judged local or self-to-landmark spatial relations and route distance. Similarly, when participants learned an unfamiliar campus building from a map or from navigation, navigation learning led to better performance on tasks assessing route knowledge, whereas map learners performed better on tasks assessing survey knowledge (Taylor et al., 1999). These findings suggested that environment knowledge differs after learning from navigation (route perspective) versus from maps (survey perspective). With a route perspective, we either consciously or incidentally acquire route knowledge including the sequential ordering of landmarks, landmark locations in relationship to the learner, and landmark appearance. When learning from a survey perspective, we gain information about global, configural relationships between landmarks (Buchner & Jansen-Osmann, 2008; Thorndyke & Hayes-Roth, 1982).

Learning from navigation versus maps also affects the orientation taken with respect to the environment. When navigating, people reorient with each turn, but with maps the map generally remains in the same orientation. This difference affects the orientation specificity of the spatial mental model. Knowledge representations acquired from navigation do not typically demonstrate fixed orientation effect; however, representations garnered from maps have been shown to be orientation-dependent (Evans & Pezdek, 1980). In the real world, the orientation difference in memory may impact the information one seeks when feeling lost or disoriented.

Studies also found different navigation performance after learning from route and survey perspective. For example, Brunyé, Gardony, Mahoney, and Taylor (2012) asked participants to study a large-scale virtual environment from different spatial perspectives. Route perspective learning better supported local or proximal navigation, whereas survey perspective learning better supported navigation between locations more distant from one another. Taken together, these findings suggest that the perspective taken during learning affects the spatial representation that develops (Buchner & Jansen-Osmann, 2008; Thorndyke & Hayes-Roth, 1982).

Most EL studies exploring how spatial information source and perspective impact spatial knowledge—which includes representations of route, survey, and landmark information—limit learning to a single perspective. However, real-world EL is usually not restricted to one perspective. We suggest that multiple perspectives may not only facilitate EL but may also be a preferred metacognitive strategy. Because survey and route perspectives provide different environment information, learners taking one perspective may strategically seek information from the other perspective, particularly if they are monitoring their own learning process or run into unexpected information (e.g. a detour or a landmark in a location different from their expectation). Therefore, when given access to both perspectives, e.g. using a smartphone when navigating, learners may strategically control learning by switching perspectives, in a goal-directed manner. For instance, when the study goal is to simply navigate to a destination, learners may switch less frequently as compared with when they need to remember the layout for later retrieval.

Modern technology increasingly allows easy perspective switching during EL. However, most studies in spatial cognition have explored the nature of EL within perspectives independently, thereby disallowing metacognitive control over perspective selection. So far, less work has explored learning when switching or combining route and survey perspectives, especially when participant control is allowed.

To date, most of the work examining perspective switching in EL has focused on perspective differences between study and test. These studies consistently show better performance when the perspective tested matches the study perspective (Brockmole & Wang, 2003; Diwadkar & McNamara, 1997; Pazzaglia & Taylor, 2007; Shelton & McNamara, 1997, 2004; Wang Q (2013): Perceptuo-motor associations in spatial knowledge: Encoding or retrieval effect?, unpublished). Consistent with *Transfer Appropriate Processing* (Morris, Bransford, & Franks, 1977), participants who study an environment by navigating tend to perform better on tasks assessing route knowledge, such as navigating between landmarks (Brunyé et al., 2012; Pazzaglia & Taylor, 2007). Map-learners show better performance on survey tasks, such as estimating Euclidian distances (Brunyé et al., 2012; Thorndyke & Hayes-Roth, 1982; Taylor et al., 1999). When the tested perspective differs from the studied perspective, there is usually a cost to performance (Brockmole & Wang, 2003; Taylor et al., 1999; Thorndyke & Hayes-Roth, 1982). These findings are consistent with the metacognition literature, which suggests that expectation about an upcoming test impact learning (Thomas & McDaniel, 2007). Learners who expect one type of test (recall or recognition) encode information consistent with this expectation (cf. Neely & Balota, 1981). In addition, when given a choice between recall or recognition as a form of self-testing, participants choose to study in a manner consistent with the expected test (Kang SHK (2009): The influence of test expectancy, test format, and test experience on study strategy selection and long-term retention, unpublished). Therefore, anticipation or knowledge of how spatial information will be used might impact learning strategies, including the perspective studied.

**Fig. 2:**
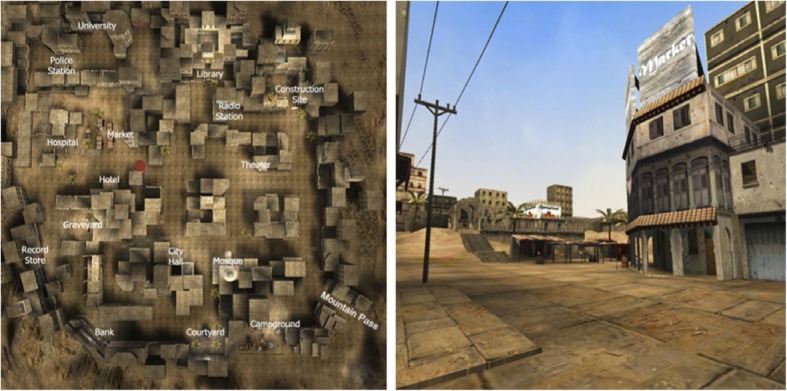
The combination of survey and route display in Brunyé et al. (2012)

**Fig. 3:**
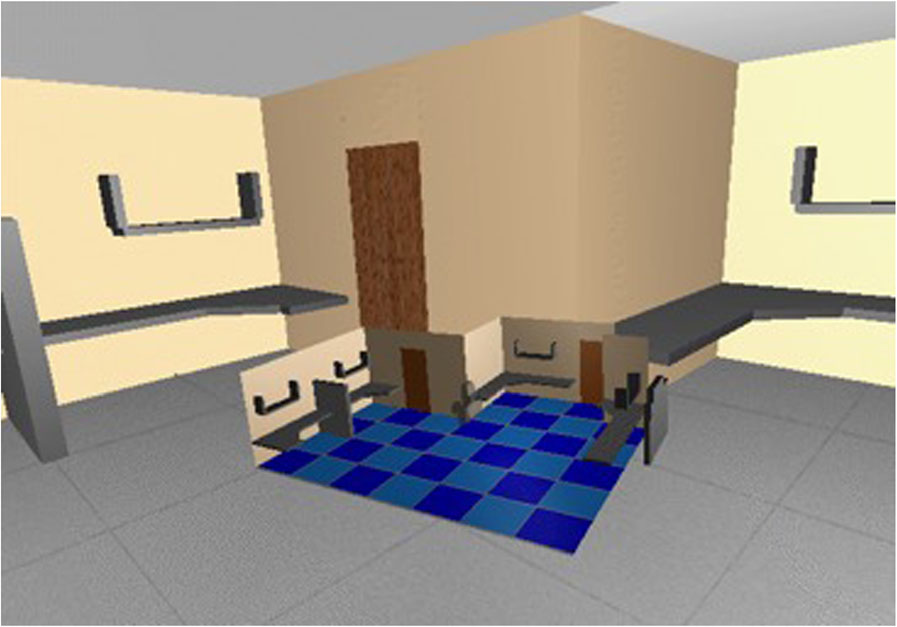
An example WIM displayed environment in Stoakley et al. (1995)

Few studies have explored learning from combined, or alternating between, route and survey perspectives. One study asked participants to learn an unfamiliar large-scale virtual environment from a video showing either one or both route and survey perspectives (Fig. 2 shows a split-screen display with both perspectives; Brunyé et al., 2012). They found that having both perspectives did not improve performance compared with the single perspective conditions. However, using a world-in-miniature (WIM) display (Fig. 3), researchers demonstrated that access to varied perspectives resulted in better environmental learning (Darken & Cevik, 1999; Ruddle, Payne, & Jones, 1999; Stoakley, Conway, & Pausch, 1995). The WIM display, typically used in first-person video games, shows a smaller top-down view (i.e. a map with the updated position of the learner) somewhere within a full-screen route perspective display. Importantly, in both Brunyé et al. (2012) and the WIM studies, perspective switching was neither forced nor directly measured. Showing both perspectives simultaneously did not ensure switches between the perspectives. Thus, it remains unknown whether participants switched or not, and if they did switch, how often and when switching happened.

The few studies that have explicitly explored the effect of perspective switching during learning have mixed findings. For example, using verbal spatial descriptions, Lee and Tversky (2001, 2005) explored the effect of perspective switching during learning on reading times and comprehension. Participants studied a paragraph describing an environment either from a route perspective, using terms of a viewer-centered reference (e.g. “left/right”), or a survey perspective, using object-centered reference terms (e.g. cardinal terms like “west/east”). The paragraph’s last sentence was either from the same (non-switching) or the opposite perspective (switching). Costs in the form of increased reading times and response times and decreased accuracy were found in both survey-to-route and route-to-survey switching conditions. Additionally, switching from route-to-survey appeared more difficult (Lee & Tversky, 2001, 2005). Such asymmetry in perspective switching demands may affect how learners monitor and control the way they gather spatial information.

**Fig. 4:**
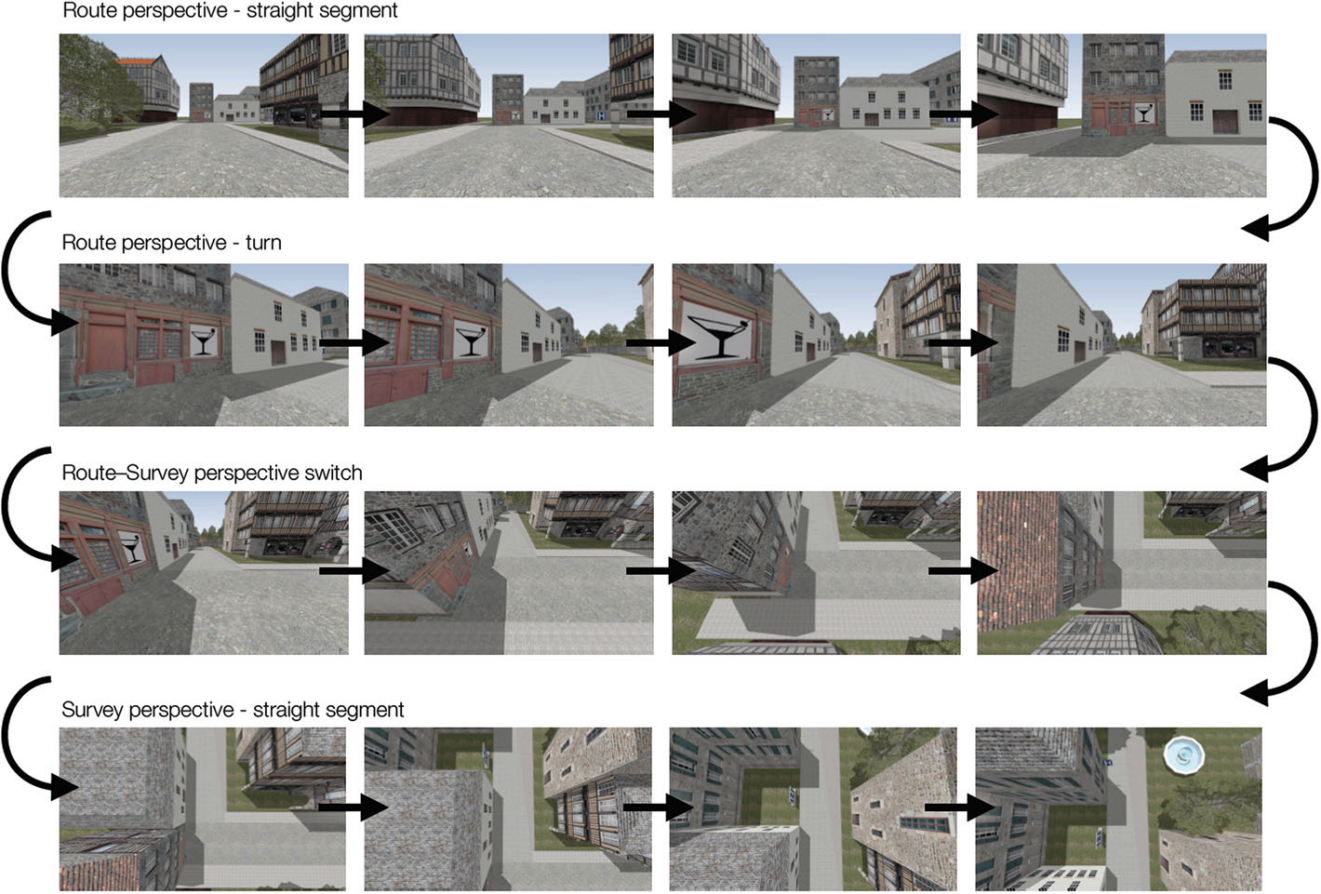
An example switch from route to survey perspective in (McGahan, M. (2014). Perspective switching in virtual environments. Unpublished doctoral dissertation, Columbia University. https://academiccommons.columbia.edu/doi/10.7916/D8R20ZGD)

A recent investigation of perspective switching gave participants the opportunity to visually switch perspectives during virtual environment learning (McGahan, 2014). The study aimed to examine how switching perspectives during study affected memory of the environment (Fig. 4 provides an example of a route to survey switch). In Experiment 1, participants were forced to switch once, either from route to survey or the reverse. In Experiment 2, participants could control switching. It was found that forced switching did not improve memory for landmarks and their locations. However, self-controlled switching improved recognition and recall of the landmarks and location information, without increasing total study time. This suggests that metacognitive control may facilitate environment learning.

### The role of metacognitive control in EL

While the role of metacognitive control in EL has received little attention, the few relevant studies suggest its potential importance. Further, previous research has shown that factors influencing metacognitive control also affect EL. As one example, motivational factors such as learning goals and outcome expectations affect how learning is regulated and influence the information represented in memory after EL. Previous findings suggest that having a mastery goal (e.g. to build up skills or knowledge of the studied materials) affects study time allocation (Son & Metcalfe, 2000; Thiede & Dunlosky, 1999). Other goals have also been observed to affect how learners regulate their learning. For instance, participants may choose to study easy items or prefer easy tasks, when: (1) the goal is externally oriented, such as to maximize reward or show their success (Wolters, 2003); or (2) participants want to expend less effort on a task, or reduce the cost associated with a task (Goldsmith, Koriat, & Pansky, 2005; Thorkildsen & Nicholls, 1998).

For EL, goals affect representations developed from maps and navigation (Gauvain & Rogoff, 1986; Magliano, Cohen, Allen, & Rodrigue, 1995; Taylor et al., 1999). In EL, goals are often induced through instructions. A route goal may ask participants to learn the fastest routes between locations while a survey goal may ask participants to learn the environment layout. These instructions impact performance in a perspective consistent way (Magliano et al., 1995; Taylor et al., 1999). More interestingly, learning format (e.g. navigation versus map) and learning goal interacted. Map learners encoded route information, when directed by a route goal; similarly, navigators encoded survey information when directed to study a building layout. Therefore, learning goals can play an important role in spatial representation development, possibly by directing learners’ strategic selection over different sources of spatial information (Shelton & McNamara, 1997, 2004; Taylor et al., 1999; Wang, 2013). Prior experience or pre-existing knowledge also affects metacognition and EL. Metacognition studies suggest that pre-existing knowledge impacts metacognitive control, possibly by influencing participants’ monitoring process (Frank & Kuhlmann, 2017; Mueller & Dunlosky, 2017; Mueller, Dunlosky, & Tauber, 2016). According to Koriat (2000), pre-existing knowledge about the learning, and knowledge about the learners themselves, may jointly influence how learners monitor and control the learning process. For example, knowing how well one has mastered vocabulary for the Scholastic Assessment Test (SAT), and what strengths he may have as a learner (e.g. he may be good at remembering words), could lead to different judgments of the learning process and, in turn, affect how he studies or restudies vocabulary for the Graduate Record Examinations (GRE). Research has also explored how meta-strategic knowledge affects decisions about which strategies to use. Learners who possess knowledge about potentially useful learning strategies can select strategies to facilitate learning (Pressley, Levin, & Ghatala, 1984; Reder & Ritter, 1992). For example, an instructor may regularly use retrieval practice to learn the names of students in her class. Taken together, these findings suggest that individuals’ knowledge and/or experience affects metacognitive judgments-making, which could, in turn, impact regulation.

For EL, experience with an environment can influence learning from different spatial perspectives (Brunyé et al., 2012; Brunyé & Taylor, 2008; Pick & Lockman, 1981; Thorndyke & Hayes-Roth, 1982). With more exposure to an environment, people tend to develop a more survey-like representation. For example, more experienced taxi drivers demonstrate better survey knowledge compared with their passengers (Appleyard, 1970; Maguire, Woollett, & Spiers, 2006). In addition, with moderate exposure to an environment, map-learners were better than navigators in estimating Euclidian distances and making relative location judgments; however, this map-learning advantage disappeared with increased environment experience (Thorndyke & Hayes-Roth, 1982). Similarly, Brunyé et al. (2012) asked participants to study an unfamiliar virtual environment from a video. Participants’ knowledge of the environment was examined by navigating between 10 successive landmarks. The slope of improvement in participants’ navigation performance, from Trial 1 to 10, was assessed. They found that accumulated navigation experience, gained over the course of 10 successive trials, supported spatial memories of the routes, landmarks, and overall layout of the environment. These knowledge gains, in turn, enabled mental perspective-switching to access information needed for the navigation task. In other words, the spatial representations allowed for greater cognitive flexibility. Such flexible representation could efficiently guide route planning (detours). In addition, they found that cognitive load for learning from different perspectives may be asymmetric. Compared with a survey perspective, learning an environment from a route perspective might require more time to reach the same levels of representational flexibility (Brunyé et al., 2012). This finding has implications for metacognitive control in EL. Those taking a route perspective may invoke different learning sources (i.e. maps on cellphones or GPS devices), if available, thus taking greater metacognitive control over their environment learning. This may also suggest that, to be able to flexibly navigate between locations, learners may switch perspectives more often when learning from a route perspective than when learning from a survey perspective.

Finally, previous studies suggested that self-efficacy beliefs affect metacognitive control in learning (Linnenbrink & Pintrich, 2003; Zimmerman, Bandura, & Martinez-Pons, 1992). For a learner to successfully use potential strategies, knowledge about the strategies alone is not enough; the learner must believe that he/she can use the strategies. Previous literature exploring the effect of self-efficacy beliefs on learners’ use of strategies has found positive relationships between the two. High self-efficacy relates positively to more extensive use of cognitive strategies, such as strategically organizing, planning, and monitoring as well as controlling of study process (Bandura, Barbaranelli, Caprara, & Pastorelli, 1996, 2001; Bandura, Caprara, Barbaranelli, Gerbino, & Pastorelli, 2003; Zimmerman & Bandura, 1994). For example, individuals with high self-efficacy demonstrate more effective monitoring over the learning process, suggesting that self-efficacy does affect metacognitive processes (Pajares, 2008). Additionally, self-efficacy may relate to other factors such as learners’ interest or value of the task (Linnenbrink & Pintrich, 2003), which may also lead to more engagement in studying and better learning (Pintrich & Schunk, 1996).

In the context of EL, individuals vary widely in strategy preference and ability. Studies have found individual differences in route or survey knowledge development (Ishikawa & Montello, 2006; Wolbers & Hegarty, 2010) and in perspective preference during learning (Hegarty, Richardson, Montello, Lovelace, & Subbiah, 2002; Pazzaglia & De Beni, 2001). Participants report different preferences for spatial perspectives, which may further affect how they study unfamiliar environments (Pazzaglia & De Beni, 2001). People also differ in the strategies used when studying maps or navigating (Baldwin & Reagan, 2009; Kozlowski & Bryant, 1977; Pazzaglia & De Beni, 2001). Baldwin and Reagan (2009), for example, found that individuals with a good or bad self-reported sense of direction (SOD) differed in strategies used in learning. Poor SOD individuals relied more on verbal resources, whereas good SOD participants relied more heavily on visuospatial information. These findings indicate that differences in preference and spatial abilities could affect a learner’s strategic choice of information source when learning an environment. A learner’s self-efficacy or preference may affect how long they choose to study from a particular perspective. Therefore, research into how self-efficacy and strategy preference impact metacognitive processes could directly inform how learners exercise control over EL.

The aforementioned factors (goals, experience, or knowledge of the environment, preference and self-efficacy beliefs) are important in the context of the current review, because these factors may individually, or interactively, contribute to how learners control their learning of unfamiliar environments. Although not directly examined in those studies, the aforementioned factors seem to influence learning by affecting learners’ choice over strategies or different sources of information, therefore suggesting that metacognitive control may play an important role in learning unfamiliar environments.

However, we should note that although there are possible relationships between metacognition and EL, that metacognitive control may be essential in how learners study new environments, there is, nevertheless, a gap in the literature that directly examines the effect of metacognitive control over perspective switching in EL. Whereas the influential factors have been demonstrated to affect metacognitive control in verbal learning, how these factors influence the way learners control study of unfamiliar environments calls for direct exploration. Additionally, compared with traditional metacognitive research in verbal learning, dynamic EL may pose new problems. As one example, when studying new environments, people may switch between different spatial perspectives, which usually involve learning from multimedia environments. For instance, when studying a new environment through driving, we may have access to a GPS device that could provide both visual (maps) and auditory (verbal instruction) information. We may, at the same time, get instructions from other sources, such as a passenger who is familiar with the environment. Studying new environments in the real-world, therefore, may involve learning from different sources, sometimes from multimedia situations. To provide some background information in existing literature that addresses this issue, we discuss, in the next section, research and findings exploring learning in more complex multimedia environments.

### Learning in interactive (multimedia or hypermedia) environments

First introduced by Ted Nelson (1965), a multimedia or hypermedia learning environment is an open-ended environment that enables learning from multimedia resources, including video, audio, graphics, and texts (Burton, Moore, & Holmes, 1995). In such interactive learning environments, participants are usually given more control over their study process than single media learning sources. For example, they can manipulate how information is presented and can interact with these representations in different ways to facilitate learning (Azevedo, 2005; Lawless & Brown, 1997; Scheiter & Gerjets, 2007). With the boom in technological development, learning from highly interactive multimedia or hypermedia environments has become commonplace. Although previous research has not focused on multimedia EL, a brief review of multimedia/hypermedia learning results can provide insights into how cognitive and metacognitive functions may be affected in complex learning situations.

In general, research exploring learning in interactive multimedia/ hypermedia environments has yielded mixed results. Some studies suggest an advantage of being able to manipulate the information and representations. This self-controlled exploration facilitates learning and knowledge transfer, possibly by drawing students’ interest, heightening self-efficacy, and providing more constructive information processing (Jacobson & Spiro, 1995; Kozma, 1991; Rouet & Levonen, 1996). In contrast, studies also suggest that unrestricted interactions with multimedia/hypermedia systems without proper guidance may hinder learning (Kozma, 1991; Scheiter & Gerjets, 2007). The complexity of such systems may leave students feeling disoriented in hyperspace (Astleitner & Leutner, 1995; Foss, 1989), distracted by irrelevant information (Anderson & Lebiere, 1998), and suffering from cognitive overload (Conklin, 1987; Niederhauser, Reynolds, Salmen, & Skolmoski, 2000; Scheiter & Gerjets, 2007).

Findings of how hypermedia/multimedia learning environments affect metacognitive processes have also been mixed. Research has shown that structured hypermedia environments can foster metacognitive abilities and skills (Azevedo, 2005; Azevedo, Johnson, Chauncey, & Burkett, 2010). For example, Azevedo and colleagues embedded adaptive scaffolds in their hypermedia learning systems that prompt students to monitor learning and progress towards the study goal. These structured hypermedia environments successfully fostered students’ metacognitive skills (see Azevedo, 2005 for an overview). Conversely, cognitive overload and disorientation caused by such learning environments may hamper metacognitive monitoring and subsequently control over learning (Niederhauser et al., 2000; Scheiter & Gerjets, 2007). For instance, using hyperlinks extensively likely consumes cognitive resources, making it harder to strategically choose which content to read and/or determine an effective reading order (Niederhauser et al., 2000).

Taken together, hypermedia/multimedia environments can successfully promote learning if used in a structured way. An important message from this for EL is that learners should have access to metacognitive and cognitive skills to cope with the processing demands complex environments pose (Scheiter & Gerjets, 2007). For example, studies examining perspective switching in EL should consider the demands of highly interactive environments, to avoid disorientation, distraction, and cognitive overload. Many multimedia technologies designed to facilitate real-world EL, such as a GPS device providing visual and auditory information, should consider presenting information in a way that reduces cognitive load. Doing so could lead to cognitive and metacognitive benefits.

### Perspective switching in real-world environment learning

People engage in the complex task of learning new environments regularly. We study environments as pedestrians, as drivers, and/or with maps. Although people take different approaches to real-world EL, goals they have for learning the environment likely play a role. For instance, when learning a new environment, a pedestrian and a driver may form a more route-based mental representation to meet their goal of getting to a destination. In contrast, a soldier who needs to learn an unfamiliar battlefield, may develop a representation of the environment with both first-person route perspective and overall survey perspective information. Having two types of information would allow the soldier to both follow a particular mission plan and undertake an unplanned retreat.

Regardless of how new environments are learned, modern technology allows real-world learners to access different spatial perspectives. A pedestrian may access a map on a smartphone; a driver in an unfamiliar environment may rely on a GPS device with a map. When given the chance to freely control how to study or explore an environment, people with these technologies can easily switch between perspectives.

Because different learners have different learning goals, a better understanding of whether and how learners may strategically control perspective taking seems important to understand and facilitate real-world EL. On one hand, the rapid development of modern technologies has made the question more salient, because switching between different perspectives has become much easier. On the other hand, a deeper understanding of the mechanisms underlying real-world perspective-switching can provide practical guidance to technology design, such as navigational aids that provide the information and switch perspectives based on navigators’ need. However, studies to date have largely not examined metacognitive control in EL, much less whether learners with different goals differ in approaches to perspective switching. Therefore, the current review calls for further exploration of metacognition in EL, in hopes of providing useful implications to the design of navigational aids to better target needs from different learners and promote environment learning.

## Future directions

As discussed, most EL research did not enable participants to freely control which information to study, therefore limiting participants’ ability to choose and/or switch between spatial perspectives. To examine how metacognition affects how participants switch between different perspectives in EL, future work should give participants control over the learning process. We now discuss a few empirical questions that may be important in advancing our understanding of metacognitive control over perspective switching in EL.

### When/where do participants switch between perspectives in EL?

Although a few studies examined whether changing perspectives affected EL, perspective switching was neither directly tested nor recorded. Therefore, it remains unknown whether, when, and where participants strategically switch during learning. Initial work should focus on this basic point. For this basic question, learners could have access to both perspectives simultaneously (similar to the Brunyé et al., 2012 split-screen video). Eye movements could then reveal whether and how many times participants switch between the perspectives. Examining purposeful switching, learners could switch perspectives on demand (akin to using a mobile device). This could be accomplished by pressing a key to switch or using available gestural commands with a Hololens.

In addition, different models of metacognition, as previously presented, may predict different switching patterns in EL. For instance, according to Nelson and Narens (1994), the way participants strategically switch between perspectives (control) should be affected by their monitoring of the learning process. In this case, the effectiveness of switching patterns would be impacted by the accuracy of the monitoring. Further, the goal of the EL task should impact control. For example, if the goal is simply to find the shortest route between point A and point B, the control processes implemented should be qualitatively different than if the goal is to remember the landmarks on a specific route. Pintrich (2000), on the other hand, may predict that in self-regulated EL, learners would actively make plans to study, which could, in turn, impact the way they gather spatial information, possibly making switching less frequently. Exploring switching patterns when learning new environments could be a way to test whether different models of metacognition can be extended to the field of EL and, therefore, provides an interesting possible direction.

### What factors affect perspective switching in EL?

The basic question above begs some immediate follow-up questions, ones that draw on influential factors shared by both metacognitive and EL processes. For example, if participants do switch between perspectives, what contexts or motivations trigger switching (e.g. environment complexity, learning goals, metacognitive monitoring, individual differences in spatial skills)? To address context, virtual environments could vary landmark similarity, intersection complexity, or field of view range. Different learning goals could change motivations for interacting with an environment. Additionally, think-aloud protocols or direct questions, such as “Why did you decide to switch to the other display?” could explore metacognitive and motivational factors impacting perspective switching. In addition, using individual difference measures, such as the Santa Barbara Sense of Direction Scale (Hegarty et al., 2002) or the Scale on Sense of Direction and Spatial Representation (Pazzaglia, Cornoldi, & De Beni, 2000) and tying these to contextual factors and metacognitive monitoring should provide insights into how these factors interact. These factors may individually or jointly affect patterns of perspective switching and the different patterns could, in turn, affect EL. Results from studies such as these can provide useful guidance for navigational aid design.

### How do monitoring and control interact in environmental learning?

Following closely on the metacognition literature, future directions should explore the relationship and interaction between monitoring and control as applied to EL. For example, verbal metacognitive studies suggest that while metacognitive monitoring may affect control (but see Koriat et al., 2006), accurate monitoring does not necessarily guarantee effective control (Efklides, 2014; Karpicke, 2009). Determining if this pattern holds for EL tasks will help to refine models of metacognition. EL primarily involves processing visuospatial information, which maps onto different underlying processes as compared to verbal information (e.g. Thomas, Bonura, Taylor, & Brunyé, 2012). It remains unknown as to whether learners are able to monitor and exercise control over processes associated with EL. As one example, learners may be less able to effectively monitor automatic processes of extracting spatial relationships that are often associated with EL (e.g. Thomas et al., 2012). Similar to the verbal metacognition work, future studies could assess the relationship between participants’ learning effectiveness ratings and their actual performance in navigation tasks or map-drawing tasks. The effectiveness of control can be determined by assessing whether participants do better when they can control the learning process themselves, compared with when they cannot, such as presenting the opposite perspective from that selected (adopted from Kornell & Metcalfe, 2006).

On the other hand, research in metacognition has suggested that control, in reverse, also affects monitoring. Metacognitive judgments, such as judgment-of-learning (JOL) and feeling-of-knowing (FOK) ratings, are based on the feedback from the control process, such as how fast or how fluent to retrieve a learned item (Benjamin, Bjork, & Schwartz, 1998; Hertzog, Dunlosky, Robinson, & Kidder, 2003; Koriat, 1993; Koriat et al., 2006). However, whether monitoring is control-based in EL has not been examined. It would be interesting and meaningful for future studies to explore whether and how switching between perspectives influences monitoring judgments such as JOL and FOK in EL. Such explorations will further extend our understanding of the role metacognition may play in EL.

## Conclusion

In this paper, we reviewed models of metacognition and different lines of research in the EL literature on perspective switching. Based on this literature, we point out an important issue: while research has examined whether changing perspectives affects EL, the metacognitive aspect of how learner controls the learning remains less well-understood. Little is known about whether, when, and where participants might strategically switch perspectives, when given control over learning. Although metacognitive control and EL share some influencing factors, what is still missing in the literature is whether metacognitive control plays a role in EL by influencing perspective switching during study. Therefore, it will be a promising future direction to bridge the fields of metacognition and EL, by directly examining how strategic control over perspective switching affects EL. Such exploration allows us to better understand the relationship between metacognition and EL and has important implications to facilitate EL and navigational aids design.

## Abbreviations

EL: Environment learning
FOK: Feeling-of-knowing
GPS: Global Positioning System
JOL: Judgment-of-learning
SOD: Sense of direction
WIM: World-in-miniature
